# Changes in Gait Metrics and Motor Strategies Post-Neurocognitive Rehabilitation in Subacute Stroke: A Retrospective Mixed-Methods Study

**DOI:** 10.7759/cureus.86264

**Published:** 2025-06-18

**Authors:** Keisuke Goto, Tsubasa Kawasaki, Hiroyuki Hamada, Vivian Sihan Lim, Ryusuke Shimada, Rumiko Nakazato, Yukari Horimoto, Yumi Ikeda

**Affiliations:** 1 Department of Physical Therapy, School of Health Sciences at Narita, International University of Health and Welfare, Narita City, JPN; 2 Department of Physical Therapy, School of Health Sciences, Tokyo International University, Kawagoe, JPN; 3 Department of Human and Engineered Environmental Studies, Graduate School of Frontier Sciences, The University of Tokyo, Chiba, JPN; 4 Department of Rehabilitation, Tokyo Women's Medical University, Adachi Medical Center, Tokyo, JPN; 5 Department of Physical Therapy, Graduate School of Human Health Sciences, Tokyo Metropolitan University, Tokyo, JPN

**Keywords:** accelerometry, gait metrics, mixed methods study, motor recovery, motor strategy, stroke rehabilitation, subacute stroke

## Abstract

Background

It is known that neurocognitive rehabilitation (NCR) improves post-stroke functional outcomes. However, systematic evaluations of changes in stride regularity and step regularity remain limited. Furthermore, a few studies have combined quantitative gait metrics with qualitative analyses of self-reported motor strategies, particularly in individuals with subacute stroke.

Objective

To examine immediate changes in key gait metrics - specifically vertical stride regularity and walking speed - as well as self-reported motor strategies following a single-session NCR intervention targeting hip and trunk proprioception in individuals with subacute stroke using a retrospective observational design.

Methods

Using a convergent mixed-methods design within a retrospective framework, we concurrently analyzed quantitative gait and qualitative interview data, which were subsequently integrated during the interpretation phase. Clinical data from 39 patients with subacute stroke were analyzed. Each participant underwent a single-session NCR intervention. Clinical records included trunk accelerometry-based gait assessments and self-reported motor strategies collected through semi-structured interviews. Quantitative gait data were analyzed as time series, and qualitative interview data were processed using text mining techniques. The findings were integrated via correspondence analysis, with patients categorized based on the percentage change in vertical stride regularity from baseline to immediately post-intervention.

Results

Statistically significant immediate changes in stride regularity (vertical and anteroposterior axes) and step regularity (vertical, mediolateral, and anteroposterior axes) were observed from pre- to post-intervention (T0 to T1), with medium to large effect sizes. Gait speed showed a statistically significant increase post-intervention to the following day (T1 to T2). Co-occurrence network analysis of interview data revealed a shift in reported motor strategies post-intervention, from distal segments (such as “feet” and “toes”) to proximal segments (such as “hip joint,” “waist,” and “trunk”). The correspondence analysis suggested that patients with greater changes in vertical stride regularity described strategies emphasizing proximal control, whereas those with smaller changes referenced distal segments or fall prevention-oriented strategies.

Conclusions

A single session of NCR focusing on hip and trunk proprioception was associated with immediate changes in gait regularity and symmetry, as well as patients’ awareness of their motor strategies in subacute stroke. The integration of objective and subjective assessments may support more personalized rehabilitation planning. Prospective studies are warranted to further investigate these findings.

## Introduction

In post-stroke rehabilitation, training approaches that emphasize the integration of sensation and motor function are widely employed to support motor recovery. Previous research suggests that combining sensory-focused training with conventional motor-oriented approaches may yield better functional outcomes [1]. However, evidence regarding the effects of sensory training remains limited, and the specific contributions of interventions such as sensory discrimination training and perceptual learning require further investigation. In particular, given that regaining walking ability is a major goal for individuals post-stroke, the relationship between sensory training and motor strategy modifications during gait remains unclear and warrants further exploration [2]. Addressing this gap requires research into how active sensory inputs are processed and integrated into movement patterns, especially in complex tasks like gait.

In response to these challenges, neurocognitive rehabilitation (NCR) has garnered attention as a practical model of active sensory training [3]. NCR encourages patients to actively reprocess sensory information and reconstruct motor strategies through cognitive tasks involving perception and attention [3,4]. This approach is based on the concept of "perception-action coupling," where perception and action dynamically interact [5]. It is hypothesized that active sensory processing by the patient facilitates more coordinated motor execution. The emphasis on active patient engagement and cognitive involvement distinguishes NCR from more passive or purely motor-focused interventions. Interventions based on NCR principles have been associated with various outcomes, including changes in muscle strength, balance ability [6], proprioception, spasticity, gait speed [7], and indicators of brain neuroplasticity [8]. These findings support the notion that NCR is an effective intervention for promoting functional recovery in post-stroke rehabilitation.

According to existing studies on NCR-based gait rehabilitation, gait speed has been the most commonly used outcome measure, whereas the consistency and rhythmicity of gait patterns remain underexplored. In stroke rehabilitation, promoting stable and symmetrical gait patterns, rather than simply increasing gait speed, is considered a crucial therapeutic goal [9,10]. Recent advances in wearable sensor technology, particularly triaxial accelerometry, have enabled more objective evaluation of gait characteristics such as gait regularity and symmetry, which were previously difficult to assess [11]. Among these measures, the autocorrelation coefficient (AC), derived from trunk acceleration data, is regarded as a sensitive indicator of the regularity of the gait cycle at different temporal scales. Specifically, as described by Moe-Nilssen and Helbostad (2004), it is possible to quantify both the regularity from one full stride to the next (termed stride regularity) and the regularity from one step to the next (termed step regularity) [12]. These metrics are associated with trunk function and balance ability in patients in the early stages of stroke, suggesting a potential relationship between trunk control recovery and changes in gait control [13]. Understanding how NCR relates to such changes could provide deeper insights into its role in rehabilitation practices.

Another challenge is that prior studies rarely integrated objective motor measures with patients' self-reported motor strategies at the group level. Given that NCR targets internal sensory and cognitive processes, it is critical to not only document motor outcomes but also explore how patients perceive and reconstruct their movements. Understanding patients' subjective experiences is essential for clarifying the nature of observed changes. Recently, mixed-methods approaches that combine objective data with subjective experiences have increasingly been advocated in rehabilitation research to optimize individualized treatment strategies [14]. In this context, it is important to capture both measurable changes in gait control and subjective reports of motor strategy changes following NCR to gain a more comprehensive understanding of its effects.

Therefore, this study aimed to investigate group-level associations between changes in key gait metrics - specifically vertical stride regularity and gait speed - and self-reported motor strategies following a single-session NCR intervention in individuals with subacute stroke. Using a retrospective observational design and a mixed-methods approach, we analyzed clinical data to explore these associations. By linking quantitative gait outcomes with patient-reported motor strategies, this study seeks to inform more individualized and mechanism-based approaches to stroke rehabilitation.

## Materials and methods

Study design

This study employed a convergent mixed-methods design within a retrospective observational framework. The study involved the secondary analysis of existing clinical data from patients who were hospitalized at Tokyo Women's Medical University Hospital and received a specific NCR intervention as part of their routine clinical care between January 2019 and June 2020. Quantitative and qualitative data, which had been routinely collected and recorded during this period of standard care, were extracted for concurrent analysis and subsequently integrated during the interpretation phase. The study protocol, including the retrospective use of de-identified patient data, was approved by the Ethics Committee of Tokyo Women's Medical University (approval number: 5711). Patients were informed about the study and consent was obtained via an opt-out procedure, allowing those who did not wish to participate to decline the use of their data.

Patients included in the analysis

The analysis included 39 patients with subacute stroke (14 women, 25 men; mean age, 64.2±11.1 years) who met the following inclusion criteria: being within two weeks of stroke onset; having a score of 3 on the Functional Ambulation Categories (FAC) [15], indicating their ability to walk 10 m under supervision; having received the specified NCR intervention, which was routinely implemented for eligible patients at our institution, during the study period; and being able to communicate orally with the researchers. Patients were excluded if they had severe higher brain dysfunction impairing the comprehension of study instructions; comorbid orthopedic conditions affecting gait; or severe sensory impairments that could interfere with gait or task performance. These criteria ensured a relatively homogeneous sample of patients in the subacute phase of stroke, with basic ambulatory abilities and sufficient communication skills to participate in interview-based assessments, from whose existing records data were extracted.

Intervention

The intervention consisted of a single session designed to address specific aspects of gait control, focusing on hip joint flexion and trunk/center of mass control during the swing phase of gait. A single-session method was used to capture immediate changes in gait control and motor strategies, reducing potential confounding effects of natural recovery during the subacute phase. This design was based on clinical observations that individuals post-stroke often exhibit delayed hip flexion during the pre-swing phase [16] and altered center of mass control during gait [17], which are associated with gait asymmetry, compensatory trunk/pelvic movements, and impaired dynamic balance. The tasks were conducted in a stable seated position to facilitate focused engagement with proprioceptive input and imagery of the swing-phase movement. While this approach does not involve actual gait, it is grounded in evidence that combining motor imagery with relevant somatosensory input can modulate nervous system excitability. For instance, it has been demonstrated that motor imagery of a specific movement, when paired with sub-motor threshold somatosensory stimulation, can potentiate corticospinal excitability more than motor imagery alone [18]. Our intervention, which similarly paired proprioceptive discrimination tasks (a form of targeted somatosensory input) with cognitive tasks related to gait, was hypothesized to engage similar neuroplastic mechanisms. The intervention lasted approximately 10 min and comprised two steps. A detailed, step-by-step demonstration of the intervention protocol is provided in the supplementary video (Video 1).

**Video 1:** Demonstration of the NCR intervention targeting hip and trunk control This video serves as supplementary material to the manuscript, demonstrating the NCR intervention protocol. The role of the therapist is portrayed by the first author, and the role of the patient is portrayed by a healthy adult model. All individuals depicted have provided written informed consent for publication of their images. Please note that this demonstration is intended to clearly illustrate the procedure and may not reflect the performance variability of an actual patient with subacute stroke. NCR, neurocognitive rehabilitation.

**Figure 1:**
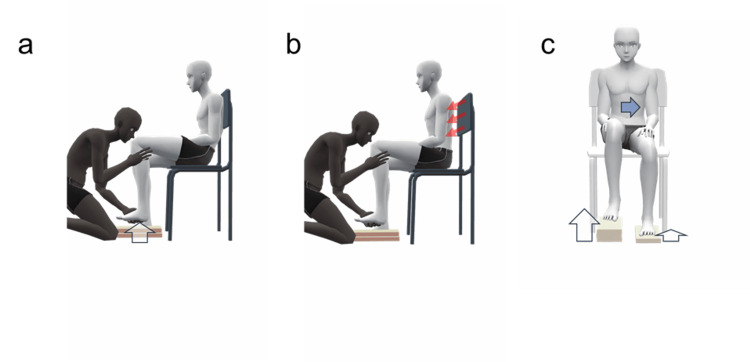
Neurocognitive rehabilitation tasks targeting trunk and hip control (a) Proprioceptive discrimination task: discrimination of hip joint flexion angles in a reclined sitting posture with back support. (b) Advanced proprioceptive discrimination task: discrimination of hip joint flexion angles while maintaining an upright trunk posture without back support, emphasizing trunk control. (c) Center of gravity recognition task: exploration and integration of weight shift awareness relevant to gait by inserting boards of different heights under each foot and perceiving subtle pressure changes at the buttocks. Blue arrows indicate center of gravity shifts; red arrows indicate conscious efforts for trunk stabilization.

The first step, hip joint flexion angle discrimination training under trunk control, aimed to facilitate the reconstruction of motor strategies by enhancing sensory processing related to hip flexion and trunk control, as a preparation for actual gait (Figure 1a, 1b). The patient sat leaning against a backrest (Figure 1a), and the therapist passively lifted the non-paretic lower limb, inserting multiple boards (made of ethylene-vinyl acetate resin) between the sole of the foot and the floor. The boards measured 300 mm in length, 110 mm in width, and 15 mm in thickness. Patients were asked to estimate the number of boards inserted. To ensure focus on bodily sensations, patients were instructed to close their eyes during this estimation task. If a patient found it difficult to maintain eye closure, they were alternatively instructed to look straight ahead and avoid looking at their feet. Therapists monitored eye gaze and provided verbal reminders as needed to minimize reliance on visual cues. The primary instruction was to focus on proprioceptive sensations related to changes in hip flexion. If the patient's estimation was correct, the therapist provided verbal confirmation and proceeded to the next trial. If the patient's estimation of the number of boards was incorrect, the therapist provided immediate feedback by adjusting the boards to the number the patient had stated, allowing them to perceive the difference, before returning to the correct number for further discrimination practice. The procedure was then repeated for the paretic limb. Subsequently, the patient adopted a seated posture without backrest support, maintaining an upright trunk (Figure 1b), and performed the same task, with the therapist instructing the patient to maintain vertical trunk alignment during limb elevation and to continue adhering to the eye closure or forward gaze instructions during board number estimation. Although this integration of trunk and hip control is typically automatic in healthy individuals, explicit training was incorporated to promote conscious control as a prerequisite for motor relearning after stroke.

The second step, a cognitive task integrating seated pressure perception and weight shift awareness relevant to swing phase initiation, aimed to encourage recognition and control of center of mass movements associated with the swing phase of gait (Figure 1c). The therapist inserted different numbers of boards under each foot to create an asymmetrical lower limb position (and thus asymmetrical buttock pressure), and patients were asked to identify the number of boards under each foot while maintaining an upright trunk and focusing on pressure sensations at the buttocks, as in Step 1. Once the patient identified the asymmetry resulting from the different board heights, the therapist provided two key verbal prompts to guide their exploration of appropriate weight distribution for gait. First, the therapist asked: "From your current leg position, which leg would you imagine swinging forward first if you started walking?". This prompt encouraged the patient to infer the more likely swing leg based on the therapist-induced lower limb asymmetry, rather than relying solely on a direct feeling of ease. Typically, the leg on the higher side (more boards) may be inferred as the one to initiate the swing. However, if the patient's perceived center of mass was not aligned with this inference (e.g., if their weight was shifted towards the higher side, making it biomechanically difficult to unweight that leg for a swing), the therapist then posed a second, more refined question: "To swing that (higher) leg, where does your center of gravity need to be for it to feel easiest?". This guided the patient to actively explore subtle weight shifts, using their buttocks pressure perception, to find a center of mass position that they felt would optimally facilitate the swing of the higher-seated leg, simulating a more biomechanically sound pre-swing weight transfer. The overarching goal was to enhance the patient's awareness and conscious control of their center of mass in preparation for the weight shifts required during actual gait.

Data collection

In this study, we used clinical data routinely collected and stored as part of patients' medical records and standard rehabilitation assessments during their acute hospital stay. The dataset included trunk acceleration data obtained during the 10-m walk test, gait speed measurements, and records from semi-structured interviews, all focused on the period immediately surrounding a specific, single-session NCR intervention that was part of routine clinical practice.

Basic Patient Information

Basic patient information was extracted from medical records, including age, sex, body mass index (BMI), stroke type and lesion site, National Institutes of Health Stroke Scale (NIHSS) score at onset, and the number of days from stroke onset to the start of the intervention analyzed in this study.

Quantitative Data: Gait Metrics

Gait metrics were assessed using the 10-m walk test at three time points relative to the NCR intervention: before (T0), immediately after (T1), and the following day (T2). This three-point evaluation captured the immediate and very short-term alterations in gait metrics associated with the single-session NCR task, utilizing data that were consistently available within the acute care rehabilitation pathway for this retrospectively analyzed cohort. Measurements included trunk acceleration and gait speed.

The walk test was performed on a 16-m walkway, with designated acceleration and deceleration zones. Patients were instructed to walk at a comfortable, self-selected speed. Trunk acceleration was recorded using a triaxial accelerometer (MG-1110, LSI Medience Corporation, Tokyo, Japan; sampling frequency: 100 Hz) attached to the center of the lower back at the level of the third lumbar vertebra. Gait speed was calculated based on the time taken to traverse the central 10-m segment.

Qualitative Data: Self-Reported Motor Strategies

Semi-structured interviews were conducted by the attending physical therapist at T0 and T1. Collecting data at these time points allowed us to capture any self-perceived changes in motor strategies associated with the intervention. With patient consent, interviews were audio-recorded and stored as part of the clinical records. The interview format consisted of two main questions: what patients consciously paid attention to while walking, and which specific body parts they were aware of and how. Supplementary questions were asked as needed to encourage deeper exploration of the patients’ statements. These open-ended yet focused questions were designed to elicit detailed descriptions of conscious motor control efforts. Interviews were conducted in Japanese to capture linguistic nuances related to self-reported motor strategy changes during gait following the intervention.

Data analysis

Quantitative data were analyzed using R (version 4.2.2, R Foundation for Statistical Computing, Vienna, Austria), and qualitative data were analyzed using KH Coder (version 3.02c) [19,20]. KH Coder is a free software for quantitative content analysis and text mining, developed and maintained by Professor Koichi Higuchi since 2001. The tool is widely used in academic and commercial settings for text analysis (https://khcoder.net/en/).

Analysis of Background Information

Patient background information, including age, sex, BMI, stroke type and lesion site, NIHSS score at onset, and number of days from onset to intervention, was extracted from medical records. Continuous variables are summarized as means±standard deviations or medians (interquartile ranges), and categorical variables as counts (percentages).

For group comparisons, independent t-tests were used for normally distributed continuous variables, Mann-Whitney U tests for non-normally distributed continuous variables, and chi-squared tests for categorical variables, with Fisher’s exact test used when expected cell frequencies were less than 5. Normality was assessed using the Shapiro-Wilk test.

Quantitative Data Processing and Statistical Analysis

Objective gait evaluation included trunk acceleration measurements and gait speed, collected during the 10-m walk test. To quantify gait rhythm and consistency, two regularity indices were derived from the trunk acceleration data using an AC analysis, following the method described by Moe-Nilssen and Helbostad [12]. Conceptually, this analysis assesses the rhythmicity of gait pattern by comparing the acceleration signal with a time-shifted version of itself. This comparison generates a function with distinct peaks, where the height of each peak indicates the degree of similarity. The second peak in this function reflects the similarity between one full stride (i.e., two steps) and the next. In this study, we quantified the height of this peak, calculated from the coefficient at the second dominant period (Ad2), as an index of stride-to-stride consistency, hereafter referred to as Stride Regularity (Ad2). The first peak in the AC function, calculated from the coefficient at the first dominant period (Ad1), reflects step-to-step consistency. As noted by Moe-Nilssen and Helbostad, a low Ad1 value can result not only from general step irregularity but also from systematic asymmetry between left and right steps [12]. Given this sensitivity to asymmetry, we utilized Ad1 in this study as a key index reflecting both step-level regularity and symmetry aspects of gait, hereafter referred to as Step Regularity (Ad1). These indices were calculated across three axes (vertical (VT), mediolateral (ML), and anteroposterior (AP)) and are denoted throughout this manuscript using this convention (e.g., VT-Ad1, ML-Ad2). Higher values (approaching +1) for both Ad1 and Ad2 generally indicate better regularity, except for ML-Ad1, where values closer to -1 reflect greater regularity [12]. These indices are widely used to capture detailed aspects of motor control during gait [21].

For AC calculation, trunk acceleration data were filtered using a 1 Hz high-pass and 20 Hz low-pass filter to remove noise and gravitational components. Ten stable gait cycles from the central section of the walkway were extracted, and Ad1 and Ad2 were calculated following the method described by Moe-Nilssen and Helbostad [12]. Gait speed was determined from the time taken to traverse the central 10 m of the walkway, with patients instructed to walk at a comfortable, self-selected pace, including acceleration and deceleration phases.

Changes in Ad1, Ad2, and gait speed were analyzed across three time points (T0, T1, and T2). Friedman's test was used to assess overall time-series changes, followed by Wilcoxon signed-rank tests with Holm correction for pairwise comparisons. For these pairwise comparisons, 95% confidence intervals (CIs) for the median of the differences were calculated using the bias-corrected and accelerated bootstrap method with 5000 resamples. Statistical significance was set at a p-value of less than 0.050. Kendall’s W was calculated as the effect size for Friedman’s test, and effect size *r* (*Z*/√*n*) was calculated for Wilcoxon tests, with magnitudes interpreted according to established guidelines [22,23]. To aid in the clinical interpretation of the observed changes, the magnitudes of change in Ad1 and Ad2 were compared against previously reported minimal detectable change (MDC) values for patients with subacute stroke, which are 0.175 (ML), 0.179 (VT), and 0.149 (AP) for Ad1 and Ad2 [24].

Additionally, to address the potential impact of sex imbalance on study outcomes, relative percentage changes in each gait metric from T0 to T2 were calculated as $\left( \frac{T_2 - T_0}{T_0} \times 100 \right)$ and compared between men and women. Comparisons were performed using independent t-tests for normally distributed data or Mann-Whitney U tests for non-normally distributed data, based on the results from the Shapiro-Wilk test.

Qualitative Data Processing and Analysis

Subjective data analysis involved co-occurrence network analysis and correspondence analysis using the KH Coder. To ensure the reliability and consensus of the findings, qualitative data processing and interpretation were conducted through a discussion among multiple researchers. Interview recordings were transcribed and tokenized into words and phrases through morphological analysis. Synonyms were consolidated, and orthographic variations were standardized to enhance analytical consistency. Co-occurrence network analysis provided an overview of changes in word usage patterns before and after the intervention, while correspondence analysis explored relationships between changes in motor performance and the characteristic vocabulary used in post-intervention narratives.

The evaluation procedures involved first examining co-occurrence relationships of words at T0 and T1. Co-occurrence network analysis was conducted on pre- and post-intervention interview texts to identify linguistic patterns related to motor strategies, including references to body parts, sensations, and attentional focus during gait. Words with a minimum frequency of six occurrences were included to reduce noise and maintain analytical focus. The top 30 co-occurring word pairs were extracted and visualized following common practices in KH Coder-based analyses, with flexible threshold adaptation according to dataset characteristics. Decisions regarding the analytical parameters and interpretation of key terms were made through discussion among co-authors with experience in qualitative research to enhance the relevance and trustworthiness of the results.

Subsequently, characteristic vocabulary and representative utterances were analyzed based on the relative change (%) in VT-Ad2. Correspondence analysis was performed on post-intervention (T1) interview texts to examine the relationships between gait performance changes and characteristic vocabulary. Patients were classified into above-median and below-median groups based on the median relative change (%) in VT-Ad2, calculated as $\left( \frac{\text{Post} - \text{Pre}}{\text{Pre}} \times 100 \right)$, selected for its reported sensitivity to temporal changes in early-stage stroke gait function [9,10]. For this analysis, words with a minimum frequency of eight occurrences were included, following the same rationale used in the co-occurrence analysis. To illustrate characteristic vocabulary, two representative utterances containing relevant words were selected for each group and verified for contextual relevance through triangulation by the fifth author (a physical therapist with clinical experience) to minimize subjective bias. Translation of text mining results and selected representative narratives from Japanese to English was performed by the fourth author, a native English speaker proficient in Japanese.

Integration of quantitative and qualitative data

In this study, changes in quantitative and qualitative data before and after the intervention were analyzed independently. Quantitative analyses focused on changes in gait metrics (such as VT-Ad2), while qualitative analyses examined shifts in patient-reported motor strategies reflected in the interview narratives.

Subsequently, to further explore the relationships between objective gait improvements and subjective changes in motor strategies, patients were retrospectively classified into groups based on the relative change (%) in VT-Ad2. Correspondence analysis was then used to examine how themes and vocabulary identified in the qualitative analysis related to the degree of gait improvement.

This approach reflects the use of a convergent mixed-methods design to provide a comprehensive understanding of the intervention’s effects by triangulating quantitative and qualitative findings.

## Results

Clinical characteristics of participants

A total of 39 patients with subacute stroke were included in this analysis. The mean age of the participants was 64.2±11.1 years. Detailed baseline demographic, clinical (including stroke type and lesion location), and pre-intervention gait characteristics, along with comparisons between male and female participants, are presented in Table 1. No statistically significant differences were observed at baseline between male and female participants for any assessed demographic, clinical, or pre-intervention gait metric (all p>0.050), suggesting comparable baseline profiles between sexes.

**Table 1:** Baseline demographic, clinical, and pre-intervention gait characteristics by sex Data are presented as means (SDs), medians (interquartile ranges), or numbers (%), as appropriate. Group comparisons were performed using the independent t-test, Mann–Whitney U test, or chi-squared tests, depending on data distribution. Fisher’s exact test was substituted for the chi-squared test when expected cell frequencies were less than 5. NIHSS, National Institutes of Health Stroke Scale; Ad2, Stride Regularity; Ad1, Step Regularity; VT, vertical; ML, mediolateral; AP, anteroposterior; 10MWT, 10-m walk test; SD, standard deviation.

Characteristics	All Participants (n=39)	Females (n=14)	Males (n=25)	Test Statistic	p-value
Age, year, mean (SD)	64.2 (11.1)	63.5 (12.0)	64.6 (10.9)	t(37)=-0.292	0.772
Body mass index, median (IQR)	22.5 (20.6–25.7)	21.1 (18.9–23.3)	22.5 (21.0–26.1)	U=109	0.053
Stroke type				Fisher’s exact test	0.446
Ischemic, n (%)	29 (74)	9 (64)	20 (80)		
Hemorrhagic, n, (%)	10 (26)	5 (36)	5 (20)		
Stroke lesion				χ²(3) = 3.128	0.372
Left cerebral hemisphere, n, (%)	14 (36)	4 (29)	10 (40)		
Right cerebral hemisphere, n, (%)	13 (33)	7 (50)	6 (24)		
Cerebellum, n, (%)	6 (15)	2 (14)	4 (16)		
Brain stem, n, (%)	6 (15)	1 (7)	5 (20)		
Time since stroke, days, mean (SD)	5.0 (3.5-6.5)	5.0 (4.0-7.8)	4.0 (3.0-6.0)	U=200	0.470
NIHSS score at admission, mean (SD)	3.0 (2.0-6.0)	3.5 (2.3-4.8)	3.0 (2.0-6.0)	U=166	0.790
Gait metrics at T0 (pre-intervention)					
VT-Ad2, mean (SD)	0.56 (0.16)	0.59 (0.15)	0.54 (0.17)	t(37)=0.955	0.346
ML-Ad2, mean (SD)	0.42 (0.14)	0.44 (0.15)	0.41 (0.14)	t(37)=0.675	0.504
AP-Ad2, mean (SD)	0.65 (0.15)	0.68 (0.15)	0.63 (0.15)	t(37)=1.02	0.314
VT-Ad1, mean (SD)	0.54 (0.16)	0.55 (0.19)	0.53 (0.14)	t(37)=0.326	0.746
ML-Ad1, median (IQR)	-0.28 (-0.40– -0.23)	-0.26 (-0.32– -0.22)	-0.34 (-0.42– -0.26)	U=223	0.166
AP-Ad1, mean (SD)	0.61 (0.16)	0.63 (0.17)	0.60 (0.15)	t(37)=0.465	0.645
10MWT speed, mean (SD)	0.71 (0.17)	0.73 (0.18)	0.71 (0.18)	t(37)=0.460	0.648

Overall changes in gait metrics

Descriptive statistics for all gait metrics at each time point (T0, T1, and T2) are provided in Table 2. Statistical comparisons of these changes over time are detailed in Table 3.

**Table 2:** Descriptive statistics of gait metrics at baseline (T0), immediately post-intervention (T1), and the following day (T2) for all participants Values are expressed as medians [interquartile range]. Abbreviations: VT, vertical; ML, mediolateral; AP, anteroposterior; SR, Stride Regularity; SS, Step Symmetry; 10MWT, 10-meter walk test.

Gait metrics	T0 : Baseline	T1 : Immediately post-intervention	T2 : The following day
VT-Ad2	0.57 [0.48-0.67]	0.67 [0.49–0.76]	0.78 [0.61–0.80]
ML-Ad2	0.40 [0.32-0.53]	0.45[0.32-0.57]	0.55 [0.41-0.64]
AP-Ad2	0.64 [0.55-0.79]	0.72[0.58-0.84]	0.73 [0.67-0.84]
VT-Ad1	0.52 [0.44-0.65]	0.60 [0.48-0.75]	0.64 [0.54-0.72]
ML-Ad1	-0.28 [-0.40 to -0.23]	-0.35 [-0.47 to -0.28]	-0.4 [-0.50 to -0.33]
AP-Ad1	0.61 [0.52-0.70]	0.69 [0.56-0.77]	0.72 [0.55-0.79]
10MWT speed	0.74 [0.59-0.84]	0.72 [0.63-0.88]	0.84 [0.71-0.93]

**Table 3:** Statistical comparisons of changes in gait metrics over time Friedman's test was used to assess overall differences across the three time points (T0, T1, and T2). Kendall’s W is reported as the effect size for the Friedman test. Pairwise comparisons were conducted using Wilcoxon signed-rank tests. P_adj indicates p-values adjusted using the Holm method for multiple comparisons; p_adj<0.050 was considered statistically significant. Effect size r is reported as the effect size for pairwise comparisons. 95% CI denotes the 95% confidence interval for the median of the difference. Abbreviations: VT, vertical; ML, mediolateral; AP, anteroposterior; Ad2, Stride Regularity; Ad1, Step Regularity; 10MWT, 10-m walk test; T0, baseline; T1, immediately post-intervention; T2, the following day. *Significant difference from T0 (p_adj<0.050). ^†^Significant difference from T1 (p_adj<0.050).

Gait metrics	Friedman Test		Post Hoc: T0 vs T1			Post Hoc: T1 vs T2			Post Hoc: T0 vs T2		
	p_adj	Kendall’s W	p_adj	Effect size (r)	95% CI	p_adj	Effect size (r)	95% CI	p_adj	Effect size (r)	95% CI
VT-Ad2	<0.001	0.254	0.033*	0.340	(-0.019, 0.088)	<0.001^†^	0.554	(0.002, 0.103)	< 0.001*	0.744	(0.068, 0.177)
ML-Ad2	0.007	0.129	0.158	0.228	(-0.046, 0.074)	0.007^†^	0.454	(-0.0001, 0.105)	0.001*	0.552	(0.024, 0.172)
AP-Ad2	<0.001	0.208	0.012*	0.431	(0.009, 0.057)	0.225	0.197	(-0.026, 0.034)	<0.001*	0.617	(0.028, 0.124)
VT-Ad1	<0.001	0.179	0.003*	0.501	(-0.0001, 0.084)	0.049^†^	0.315	(-0.007, 0.057)	<0.001*	0.666	(0.037, 0.110)
ML-Ad1	<0.001	0.366	<0.001*	0.641	(-0.089, -0.019)	0.029^†^	0.346	(-0.081, -0.008)	<0.001*	0.744	(-0.125, -0.054)
AP-Ad1	0.023	0.097	0.002*	0.503	(-0.008, 0.065)	0.557	0.096	(-0.035, 0.032)	0.001*	0.543	(0.003, 0.093)
10 MWT speed	<0.001	0.350	0.131	0.244	(-0.0253, 0.045)	<0.001^†^	0.650	(0.038, 0.135)	<0.001*	0.787	(0.057, 0.129)

Friedman's test showed significant differences among the three time points for all evaluated gait indicators (p<0.050; Kendall's W=0.097-0.366, see Table 3). Post-hoc comparisons (Table 3) between T0 and T1 revealed significant changes with moderate to large effect sizes for VT-Ad2 (p=0.033, r=0.340), AP-Ad2 (p=0.012, r=0.431), VT-Ad1 (p=0.003, r=0.501), ML-Ad1 (p<0.001, r=0.641), and AP-Ad1 (p=0.002, r=0.503). No significant changes were found at this time point for ML-Ad2 (p=0.158) or gait speed (p=0.131).

Between T1 and T2, significant changes with moderate to large effect sizes were noted for VT-Ad2 (p<0.001, r=0.554), ML-Ad2 (p=0.007, r=0.454), VT-Ad1 (p=0.049, r=0.315), ML-Ad1 (p=0.029, r=0.346), and gait speed (p<0.001, r=0.650). No significant changes were observed for AP-Ad2 (p=0.225) or AP-Ad1 (p=0.557) from immediately post-intervention to the following day.

Overall, these results indicate that multiple aspects of gait regularity at both the stride (Ad2) and step (Ad1) levels showed measurable changes over the short observation period, while changes in gait speed and ML-Ad2 became evident from T1 to T2.

Sex differences in gait metric changes

To examine whether the observed alterations varied by sex, relative percentage changes from T0 to T2 were compared between men and women. No significant differences were found for any gait parameter, including gait regularity (Ad1 and Ad2) and speed (all p>0.050).

Characteristics and gait metric changes based on VT-Ad2 alteration groups

To explore characteristics associated with the magnitude of alteration in VT-Ad2 following the NCR intervention, patients were classified into two groups based on the median relative change in VT-Ad2 from baseline (T0) to immediately post-intervention (T1): an above-median group (n=20) and a below-median group (n=19). A comparison of baseline characteristics between these two VT-Ad2 change groups is detailed in Table 4. No significant differences were observed between these groups for key baseline demographic or clinical characteristics, including age, sex, BMI, stroke type, lesion site, time since stroke, or NIHSS score at admission (p>0.050 for all comparisons, Table 4). The pre-intervention VT-Ad2 value showed a trend towards being lower in the above-median group (mean (SD): 0.51 (0.13)) compared with the below-median group (mean (SD), 0.61 (0.17)), although this difference was not statistically significant (p=0.051). The relative change (%) in VT-Ad2 was, by definition, significantly greater in the above-median group than the below-median group (p<0.001).

**Table 4:** Baseline demographic and clinical characteristics of groups categorized by median relative change in vertical stride regularity (VT-Ad2) Data are presented as means (SDs), medians [interquartile ranges, IQRs], or numbers (%), as appropriate. Group comparisons between the Above-median and Below-median groups were performed using the independent t-test for normally distributed continuous variables, Mann-Whitney U test for non-normally distributed continuous variables, and chi-squared or Fisher’s exact test for categorical variables. The groups were categorized based on the median relative change (%) in VT-Ad2 from baseline (T0) to immediately post-intervention (T1). Abbreviations: NIHSS, National Institutes of Health Stroke Scale; VT-Ad2, vertical stride regularity; SD, standard deviation.

Characteristics	All Participants (n = 39)	Above-median Group (n =20)	Below-median Group (n =19)	Test Statistic	p-value
Age, year, mean (SD)	64.2 (11.1)	63.5 (12.1)	64.9 (10.3)	t(37)=-0.401	0.691
Sex				χ²(1)=0.0144	0.900
Female, n (%)	14 (36%)	7 (35)	7 (37)		
Male, n (%)	25 (64%)	13 (65)	12 (63)		
Body mass index, mean (SD)	23.1 (4.0)	23.7 (4.4)	22.4 (3.7)	t(37)=1.03	0.310
Stroke type				Fisher’s exact test	0.160
Ischemic, n (%)	29 (74)	17 (85)	12 (63)		
Hemorrhagic, n, (%)	10 (26)	3 (15)	7 (37)		
Stroke lesion				Fisher’s exact test	0.310
Left cerebral hemisphere, n, (%)	14 (36)	5 (25)	9 (47)		
Right cerebral hemisphere, n, (%)	13 (33)	7 (35)	6 (32)		
Cerebellum, n, (%)	6 (15)	5 (25)	1 (15)		
Brain stem, n, (%)	6 (15)	3 (15)	3 (15)		
Time since stroke, days, median [IQR]	5 [3.5–6.5]	4 [3.0–6.3]	5 [4.0–6.5]	U=160	0.390
NIHSS score at admission, median [IQR]	3 [2.0–6.0]	3 [2.0–6.0]	3 [2.5–5.5]	U=167	0.520
VT Stride Regularity (VT-Ad2)					
Pre-intervention, mean (SD)	0.56 (0.16)	0.51 (0.13)	0.61 (0.17)	t(37)=-2.03	0.051
Post-intervention, mean (SD)	0.61 (0.17)	0.65 (0.15)	0.56 (0.19)	t(37)=1.64	0.112
Relative change (%)	10.3 (25.7)	29.8 (19.0)	-10.2 (12.4)	U=380	<0.001

Co-occurrence network analysis of interview data

**Figure 2:**
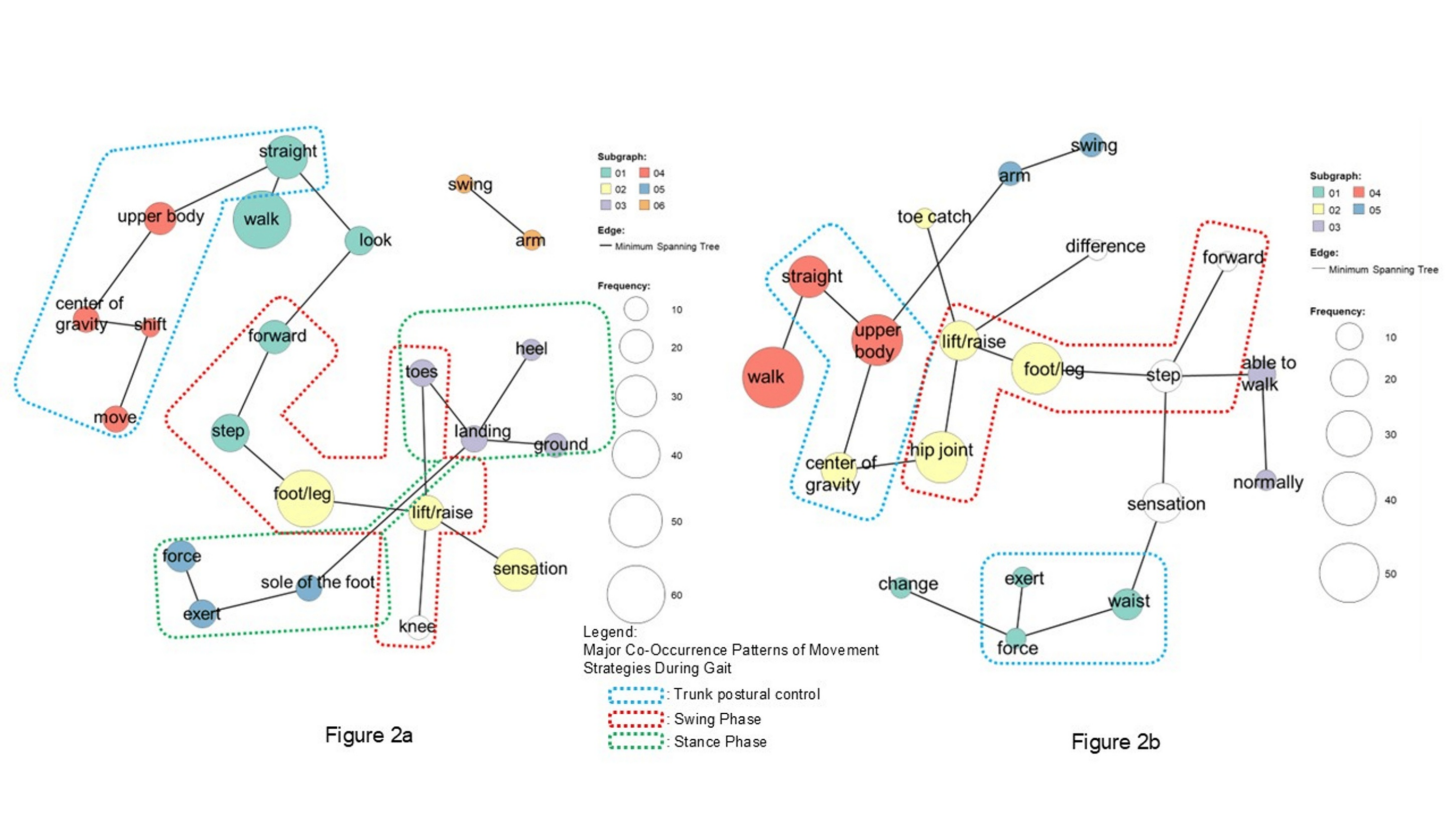
Co-occurrence networks of patient narratives regarding movement strategies during gait at pre- (a) and post-intervention (b) Each node represents a word appearing at least six times across all interviews; each edge represents a co-occurrence relationship between words. Light blue dotted lines highlight co-occurrence patterns related to trunk postural control; red dotted lines highlight patterns related to the swing phase of gait; green dotted lines highlight patterns related to the stance phase. Node size is proportional to word frequency.

Figure 2 shows the results of the co-occurrence network analysis of words related to motor strategies during gait, before and after the intervention. The analysis revealed co-occurrence patterns of words mainly related to the "swing phase," "trunk postural control," and "stance phase" during both pre- and post-intervention periods.

Regarding words related to the swing phase, at T0 (pre-intervention, Figure 2a), "lift/raise" co-occurred with words indicating distal parts, such as "foot/leg," "toes," and "knee." However, at T1 (post-intervention, Figure 2b), this pattern changed: "lift/raise" (or related concepts) co-occurred more prominently with "hip joint," indicating a shift in focus to more proximal body parts.

Regarding trunk postural control, while words such as "upper body," "center of gravity," and "straight" co-occurred at T0, words such as "waist," "force," and "sensation" also emerged at T1. This suggests that after the intervention, participants used more specific and concrete language to describe body parts and consciously engaged in postural control during gait.

Regarding words related to the stance phase, a cluster of words associated with the sole of the foot and ground contact, such as "landing," "heel," "toes," and "ground," was observed at T0. However, the frequency and co-occurrence of these terms decreased at T1.

Relationship between VT-Ad2 change groups and post-intervention (T1) narratives

**Figure 3:**
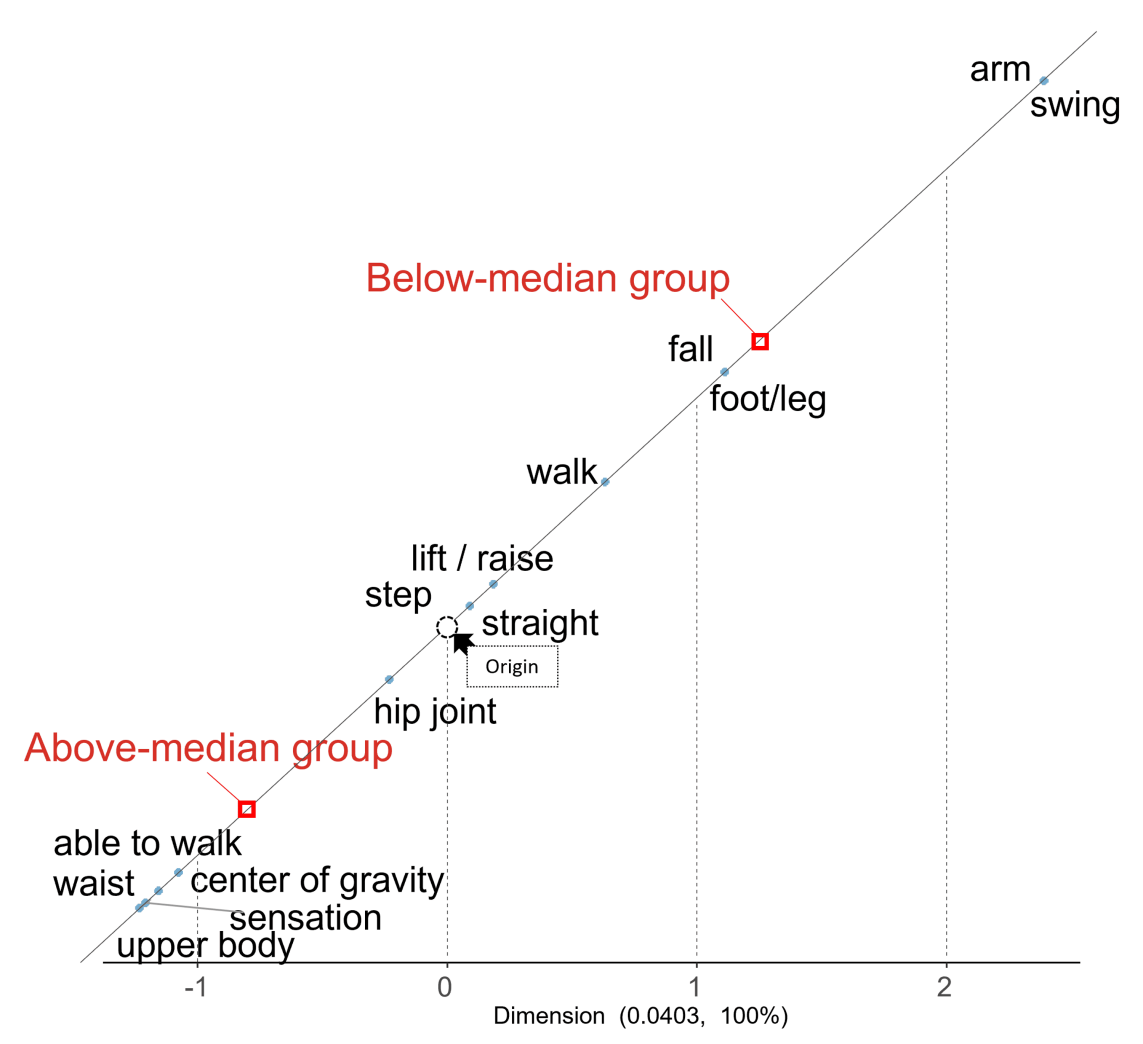
Correspondence analysis of post-intervention narratives based on VT-Ad2 change rates Red squares represent the centroids of the two participant groups classified according to the median split of VT-Ad2 change rates (Above-median and Below-median groups). The white circle represents the overall mean profile of all words. Words closer to a group centroid and farther from the origin are considered more characteristic of that group. The map is symmetrically scaled to reflect χ² distances. VT-Ad2, vertical stride regularity.

Figure 3 shows the results of the correspondence analysis performed on interview data collected immediately after the intervention (T1), comparing groups based on the rate of change in VT-Ad2. In the above-median group (characterized by greater observed alteration in VT-Ad2), words related to trunk and pelvic control, recognition of the center of gravity, and a positive sense of walking achievement, such as "upper body," "waist," "center of gravity," "able to walk," and "sensation," were prominent.

In contrast, in the below-median group (characterized by a smaller or less favorable observed change in VT-Ad2), characteristic words were primarily related to arm swing, fall prevention, and movements of the distal lower limbs, including "arm," "swing," "fall," and "foot/leg".

Table 5 presents representative statements related to the characteristic words of each group. In the above-median group, narratives often referenced proximal control and center of gravity adjustment, such as "I was conscious of keeping my upper body straight" and "I consciously moved my pelvis naturally." These were accompanied by expressions indicating a subjective improvement in walking ability, such as "I became able to walk without losing balance" and "I gained confidence in walking alone." Conversely, in the below-median group, many narratives focused on awareness of distal parts and the intention to prevent falls, as in "I was conscious of swinging my arms" and "I was conscious of not falling".

**Table 5:** Representative self-reported motor strategies in each group, associated with characteristic words identified by correspondence analysis For each group classified by the rate of change in VT-Ad2, representative self-reported motor strategies associated with characteristic words were extracted based on correspondence analysis results. Original statements, collected through semi-structured interviews in Japanese, were translated into English for presentation in this paper. VT-Ad2, vertical stride regularity.

Group	Characteristic Word	Representative Self-Reported Motor Strategies
Above-median Group	"Upper body"	"I focused more on keeping my upper body upright."
		"I walked while consciously maintaining the alignment of my upper body."
	"Waist"	"I tried to move naturally by engaging my waist."
		"I felt like I could initiate movement better by leading with my waist."
	"Center of gravity"	"I focused on keeping my center of gravity aligned while walking."
		"I consciously tried to control the movement of my center of gravity."
	"Able to walk"	"I felt like I was able to walk without losing my balance."
		"I no longer felt anxious and was able to walk by myself."
	"Sensation"	"I had a feeling that it was easier to move my legs compared to before."
		"I can sense a clear difference in weight when lifting from the hip joint as compared to forcibly lifting from the knee."
Below-median Group	"Arm & swing"	"I am able to swing my arms while walking."
		"Since I can swing my arms, I try to swing them naturally while walking."
	"Fall"	"I focused on avoiding falls to either side."
		"I consciously tried not to fall to my affected side."
	"Foot/leg"	"I focused on swinging my foot forward when walking, starting with a heel strike."
		"I focused on lifting my legs alternately when walking."

## Discussion

In this study, changes in gait regularity and symmetry were observed following an NCR intervention, and the focus of self-reported motor strategies shifted from the distal lower limbs to the trunk and pelvic region. These alterations were generally consistent across sexes. Baseline values and the magnitude of changes in gait metrics did not significantly differ between male and female participants. Participants in the above-median group - those with greater alterations in VT-Ad2 - reported more specific and intervention-aligned motor strategies. These findings suggest a potential relationship between trunk control during gait and how patients describe their movement strategies.

Immediate changes in gait regularity and symmetry were observed following a single NCR session, with a notable increase in gait speed observed the following day. Among the stride regularity metrics, only the change in VT-Ad2 exceeded its previously reported MDC value for patients with subacute stroke (0.179 for the vertical axis), with an increase of 0.21 from baseline to the following day (T0-T2) [24]. This change was accompanied by a large effect size (r=0.744), suggesting that the observed alteration in VT-Ad2 was both statistically substantial and potentially meaningful in clinical contexts. This finding suggests that the change in VT-Ad2 likely reflects a genuine change rather than measurement error, and highlights VT-Ad2 as a potentially sensitive marker of short-term motor adaptation following NCR. These changes may be associated with the intervention's focus on hip and trunk control. Previous studies reported impaired hip joint control during the swing phase [16] and altered center of mass control in patients with stroke-related gait disorders [17]; hence, this intervention could be considered a targeted approach to address these issues. However, the delayed emergence of changes in gait speed may involve multiple mechanisms. One possibility is that conscious attention to motor control increased immediately after the intervention, potentially suppressing movement automaticity temporarily. This interpretation aligns with previous reports that excessive conscious control can interfere with motor automatization in individuals with stroke [25,26]. Another possibility is that sensory information and motor strategies acquired during the intervention were integrated and consolidated during sleep, contributing to the observed gait performance alterations the following day. Sleep-dependent consolidation processes are known to enhance the performance of complex motor skills [27], and a similar mechanism may have played a role in this context. Overall, the observed alterations in gait control, including increased regularity and symmetry, suggest that even a single session of NCR may be associated with short-term changes in gait performance.

The co-occurrence network analysis of interview data indicated a shift in patients’ focus of attention from distal to proximal body regions, such as the trunk and pelvis, following the intervention. In addition, some patients described increased awareness of center of gravity control and a greater sense of confidence during walking. These observations may reflect the intervention's incorporation of tasks that emphasized internal attention, requiring patients to focus on proprioceptive feedback and subtle internal sensations. Previous research has shown that an internal focus of attention can enhance movement accuracy and motor planning under certain conditions where proprioceptive information is central [28]. The present findings are partially consistent with these reports. However, an internal focus can sometimes hinder movement automatization, with an external focus being more beneficial in other contexts [29]. Therefore, considerations regarding focus of attention should be adapted flexibly according to the patient's stage of recovery and rehabilitation goals. In this context, the findings suggest that NCR’s emphasis on internal sensory processing may be an important component influencing motor strategy development.

The mixed-methods analysis revealed that, after the intervention, increased awareness of bodily sensations critical for gait control was particularly evident in patients who showed greater alteration in VT-Ad2 (above-median group). Correspondence analysis showed that narratives from the above-median group frequently referred to proximal body regions, awareness of center of gravity control, and subjective perceptions of improved walking ability. In contrast, narratives from the below-median group emphasized distal limb control and fall prevention strategies, indicating different patterns of motor strategy awareness. These patterns suggest a potential relationship between the degree of change in motor control during gait and the nature of patients’ verbalized motor strategies. This interpretation is consistent with recent studies indicating that monitoring internal bodily sensations, including interoceptive and proprioceptive awareness, may support fine-tuning of motor performance and early detection of movement errors [30]. The present findings align with these perspectives and enhance the possibility that improved bodily awareness may be associated with refined aspects of gait control. In this context, paying close attention to patients’ descriptions of their movement strategies could offer valuable insights into the internalization of therapeutic principles and serve as a complement to objective gait assessment.

This study has a few limitations that should be considered. First, it employed a retrospective, single-center, non-randomized observational design, which limits the ability to draw causal inferences. Moreover, a formal sample size calculation was not performed, and the final sample size had a higher proportion of male participants. Hence, the study may have been underpowered to detect subtle effects, including those related to sex. Second, the short follow-up period - limited to the day after the intervention - precludes conclusions about the long-term sustainability of the observed changes. This time frame reflected the constraints of data collection in an acute care setting, where standardized longer-term follow-up was not feasible. Future prospective studies are warranted to evaluate the persistence of these effects. Third, the participants were limited to patients with subacute stroke with moderate ambulatory function (FAC score: 3), and the generalizability to patients with more severe gait impairments or at different recovery stages remains uncertain. Fourth, the interviews were conducted by the therapists who provided the intervention, which may have introduced expectancy bias. Additionally, while the NCR tasks aimed to enhance proprioceptive discrimination concerning hip and trunk control, and therapists’ verbal cues focused on task completion rather than specific segmental attention, the inherent nature of these tasks may have inadvertently guided patients' attention towards proximal body segments, potentially influencing their subsequent self-reported motor strategies. However, the use of a semi-structured interview format likely mitigated overt biases to some extent by ensuring a consistent approach to data collection and allowing for open-ended responses. Fifth, while self-reported narratives offer valuable insights, they may not fully capture nonverbal or subconscious motor adaptations such as proprioceptive adjustments. Consequently, a key limitation is the absence of objective biomechanical measurements (e.g., trunk sway, hip kinematics, surface electromyography (EMG)) to corroborate the reported shift in motor strategies from a distal to proximal focus. Consequently, definitive conclusions regarding the correspondence between subjective awareness and biomechanical change cannot be drawn. Future studies incorporating motion analysis or EMG are needed to better elucidate the neuromechanical effects of proprioceptive retraining on gait control in post-stroke populations.

## Conclusions

This exploratory study revealed that a single session of NCR was associated with immediate changes in gait symmetry and regularity in individuals with subacute stroke and may also be related to changes in subjective awareness of motor strategies. The consistency between verbalized motor strategies and gait control metrics highlights the potential value of integrating both subjective and objective assessments in gait evaluation. This study is one of the few international efforts to visualize changes in motor strategies - previously difficult to capture - through linguistic analysis. Our findings may provide a foundation for enhancing rehabilitation evaluations based on patients’ subjective experiences and for developing more individualized intervention approaches. Future prospective studies are warranted to verify the generalizability and sustainability of these observations.
